# Social network composition of vascular patients and its associations with health behavior and clinical risk factors

**DOI:** 10.1371/journal.pone.0185341

**Published:** 2017-09-28

**Authors:** Naomi Heijmans, Jan van Lieshout, Michel Wensing

**Affiliations:** 1 Scientific Institute for Quality of Healthcare, Radboud University Medical Centre, Nijmegen, The Netherlands; 2 Department of General Practice and Health Services Research, Heidelberg University Hospital, Heidelberg, Germany; Kaiser, UNITED STATES

## Abstract

**Background:**

This study aimed to explore linkages of patients’ social network composition with health behaviors and clinical risk factors.

**Methods/Design:**

This observational study was embedded in a project aimed at improving cardiovascular risk management (CRVM) in primary care. 657 vascular patients (227 with cardiovascular disease, 380 at high vascular risk), mean age 72.4 (SD 9.4) years, were recruited as were individuals patients considered important for dealing with their disease, so called alters (n = 487). Network composition was measured with structured patient questionnaires. Both patients and alters completed questionnaires to measure health behavior (habits for physical activity, diet, and smoking). Clinical risk factors (systolic blood pressure, LDL cholesterol level, and body mass index) were extracted from patients’ medical records. Six logistic regression analyses, using generalized estimating equations, were used to test three hypothesized effects of network composition (having alters with healthful behaviors, without depression, and with specialized knowledge) on six outcomes, adjusted for demographic, personal and psychological characteristics.

**Results:**

Having alters with overall healthful behavior was related to healthful patient diet (OR 2.14, 95%CI: 1.52–3.02). Having non-smoking alters in networks was related to reduced odds for patient smoking (OR 0.17, 95%CI: 0.05–0.60). No effects of presence of non-depressed alters were found. Presence of alters with specialized knowledge on CVRM was inversely related to healthful diet habits of patients (OR 0.47, 95%CI 0.24–0.89). No significant associations between social network composition and clinical risk factors were found.

**Discussion:**

Diet and smoking, but not physical exercise and clinical risk factors, were associated with social network composition of patients with vascular conditions. In this study of vascular patients, controlling for both personal and psychological factors, fewer network influences were found compared to previous research. Further research is needed to examine network structure characteristics as well as the role of psychological factors to enhance understanding health behavior of patients involved in CVRM.

## Introduction

Cardiovascular disease (CVD) was the most common cause of death for women, and the second cause of death for men, in the Netherlands in 2013 [1]. Cardiovascular risk management (CVRM) aims to prevent or delay CVD and, amongst others, heavily emphasizes control of clinical risk factors (blood pressure, serum cholesterol, body-mass index) and healthful behaviors (healthful habits for diet, physical activity, and non-smoking) [2]. Accordingly, patients have a central role in CVRM. Changing unhealthful behavior, or maintaining healthful behaviors, does not come easily. Research showed that health behavior is not only influenced by individual characteristics, but also by the individuals’ social environment. For instance, research indicated that particular aspects of social networks, e.g. high social support and social integration, were related to reduced mortality from diverse causes [3]. Subsequent studies found that persons with particular health related behaviors and characteristics tended to be connected within social networks. Such clustering patterns have been described for smoking, alcohol use, aspirin use, health screening, obesity, and depression [4–10].

So, social network studies provided compelling results which may help to understand and enhance health behaviors. The current body of evidence largely comprises of studies on social support in specific populations (including vascular patients) on the one hand and studies on social networks in general populations on the other hand. Although most studies controlled social network influences for a variety of individual characteristics (e.g. age, sex, education), few studies on health behavior used psychological traits as control variables. However, psychological traits, e.g. depression or patient activation, are known to influence health behaviors as well [11] and we were interested in the influence of social networks over and above these traits. Also, most social network studies relied exclusively on patient-reported health behaviors, which may be subject to bias, rather than recorded clinical indicators. Finally, a substantial number of studies used data of contacts of patients, so called alters, as reported on by patients, instead of including alters in the research themselves. Our research aimed to overcome these limitations of previous studies.

### Network-related factors

Several mechanisms through which social networks influence health behaviors and health outcomes have been described and include, amongst others, social support, social capital, and social influence [12]. Social support is the provision of information, practical help, or emotional comfort by individuals or organizations in the individual’s social network. It is related to improved health behavior by means of assistance with health related activities and with maintaining healthful behaviors. Provision of support can come from anyone within a given network, although evidence suggests that family seems to be most relevant for self-care [13,14].

Social capital is a related concept as it indicates the availability of support for a specific individual. Access to resources has mostly been studied by studies on social capital, defining this construct as membership in social networks that facilitate access to resources, e.g. information on health and behaviors [15]. Greater social capital has been linked to better health or well-being [16].

Social influence is a different type of mechanism in networks. The finding of clustering of behaviors (e.g. smoking, alcohol use) within networks led to the notion of social contagion of behaviors and ideas in social networks. Social contagion is a multifaceted process, which may apply to information, ideas, behaviors and infections. Multiple underlying mechanisms of contagion can result in spread of information and (resulting) behavior, e.g. imitation of successful behavior, role modeling, social comparison, and selection of contacts. In this context, homophily (also termed homogeneity) refers to the principle that contacts between persons who share similarities will occur at a higher rate than among persons who are more dissimilar [17], thus shaping opportunities for spread of information and behaviors within networks, with consequences for the formation of attitudes and norms [17,18], and social influence processes (e.g. social reinforcement) [17,19]. Noting that it is difficult to distinguish selection and causal effects [20] clustering seems to occur together with homophily.

**Fig 1:**
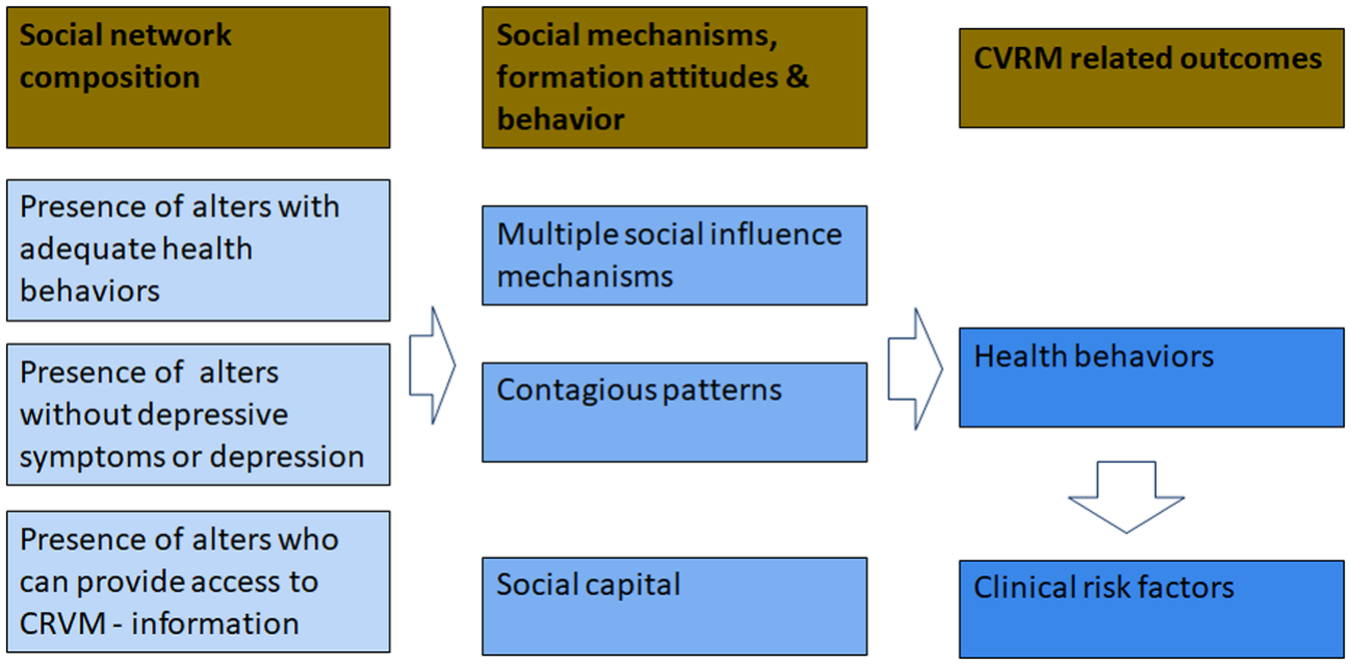
Hypothesized relations.

Using these concepts, a number of network-related determinants of health-related behaviors and clinical indicators were formulated for this study (see Fig 1 for a summary). First, as clustering is found for several behaviors and traits and can shape several opportunities for various social influence mechanisms, we expected patients to be more likely to hold healthful behaviors, that is healthful habits for physical activity, diet, and smoking, if their alters have such behaviors as well.

Second, we considered the influence of depression, which is a known predictor of many health-related behaviors. Depression can impede efforts for improving health behaviors and has a common occurrence in cardiac patients [21]. Depression and depressive symptoms have been related to impaired health behaviors and increased mortality in specific cardiac patients groups [22]. In addition to these negative effects, depression has been shown to have a contagious pattern in networks [6]. In this way, depression or depressive symptoms may assert a negative influence on health behaviors in two ways: first by influencing patients themselves and second by contagiously spreading within social networks. Therefore, we expect that social networks without individuals with depression are positively related to healthful behaviors.

Third, in addition to opportunities for spread of information and behaviors, it is obviously important that reliable information and knowledge on CVRM and health behaviors spreads within networks. Individuals who can allow for this include health professionals, such as nurses, physicians and allied health professionals. Having such persons within ones network can add to so called social capital [23], which is in this way defined as having social networks that facilitate access to resources [15]. We expect that patients with health professionals within their networks will be more likely to have healthful behaviors and have positive clinical indicators.

In the study presented here, we focused on composition of support networks of patients with high vascular risk and vascular diseases. Lifestyle support networks were constructed on individuals that patients considered to be important for managing their health-related behaviors. This definition of a social network is broader than often applied. We examined a broad, instead of specifically and narrowly, defined network for several reasons. First, we considered the result that support can be provided by anyone [24], which indicates that support for health behavior may stem from multiple specific networks (e.g. from family or friends). Second, network characteristics as identified in our hypotheses may occur in multiple networks a person engages in, e.g. alters with healthful behaviors may be a friend from a sport club or a spouse. Third, multiple types of specific networks may contribute to health.

In summary, the main aim of this research was to explore social network composition and its associations with health behaviors and clinical risk factors in patients with vascular conditions. We set out to test the following key hypotheses: Patients will be more likely to have healthful behaviors and reach target values for clinical risk factors if they have social networks which contain:

1. Individuals with healthful behaviors
2. No individuals with depressive symptoms or depression
3. Individuals with specialized knowledge on health, particularly health professionals

## Methods

### Design & study population

This study is part of the ‘Tailored Implementation for Chronic Disease’ (TICD) project [25] and was an observational study on social networks of vascular patients and their alters: individuals who patients considered important for managing their health behaviors [26]. This study was performed parallel to a larger two-arm cluster randomized controlled trial (RCT) (NTR4069). The trial aimed at testing a tailored intervention for improving CVRM in primary care by enhancing professional performance of practice nurses and included a random sample of general practices from several geographical areas in the Netherlands. Specific details of the trial are described elsewhere [27].

Patients at high risk for CVD and patients with established CVD were included. They were identified from the baseline measurement of the trial which used International Classification of Primary Care (ICPC) codes to extract eligible patients from medical records from general practices. Extraction was performed by practice nurses in cooperation with research assistants. Eligible patients were 18 years or older and capable of providing informed consent; exclusion criteria consisted of: diabetes mellitus, pregnancy and lactation, terminal illness, cognitive impairments, and poor language skills. Patients with diabetes were excluded using ICPC codes, practice nurses assessed other exclusion criteria. Alters of patients consisted of individuals that patients indicated to be important for managing their health behaviors. A maximum of four alters was included as literature indicated this is the maximum number of important or significant others to be expected within social networks of patients [28].

### Ethical approval

The Medical Ethical Committee of Radboud University Nijmegen Medical Centre has waived approval for the social network study [26] and the RCT [27]. The study protocols and all its materials (e.g. informed consent forms, questionnaires and letters), as well as the consent process, for both studies were submitted to the Medical Ethical Committee of Radboud University Medical Centre Nijmegen. This committee assessed that the Dutch law for medical scientific research does not apply to these studies. As the studies did not involve testing of body materials, no approval was required from a local medical ethical committee [S1 and S2 Files]. Participants of this study provided consent by signing written informed consent forms. All data were collected prospectively, and consisted of questionnaire data on social networks and health behavior of patients, questionnaire data on health behavior of alters, and data extracted from medical records of patients on clinical risk factors (systolic blood pressure and LDL cholesterol) and professional performance of practice nurses. None of the authors were treating physicians of participants in the social network study and the RCT study.

### Data collection procedures

**Fig 2:**
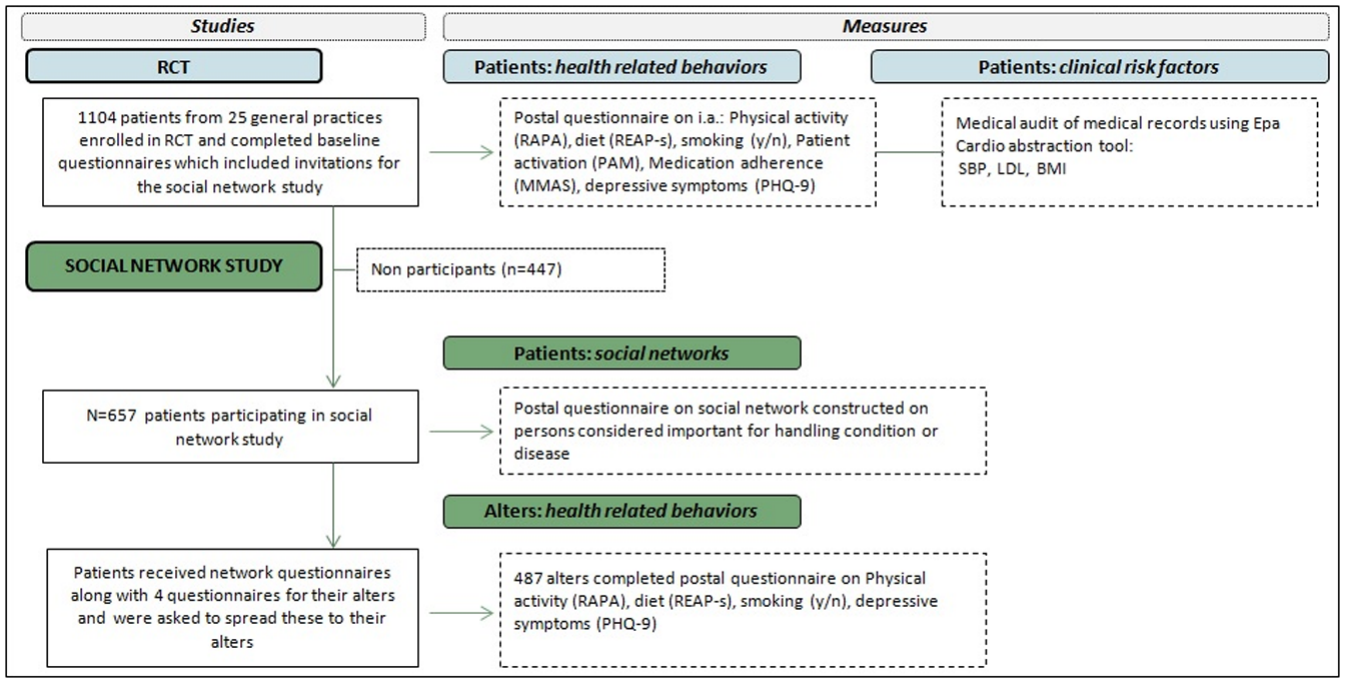
Study flow.

Patients were invited for participation in the social network study using invitations included at the end of postal questionnaire booklets send for purposes of the trial at baseline of its intervention program (see also Fig 2 ‘study flow’). Postal questionnaires for the RCT mainly focused on health related lifestyle. Invitations for the social network study contained a concise explanation on the study purpose and were accepted by completing an enclosed informed consent form. Postal questionnaires for the social network study [S3 File] were send up to a maximum of three months after receipt of completed informed consent forms. This interval was needed due to logistical constraints in the RCT. Data collection was performed from December 2013 until March 2014. For including alters of patients, four additional questionnaires titled ‘questionnaire for close ones’ were send along with patients questionnaires. These four alter questionnaires had identical contents. An information letter was used to inform patients that these questionnaires were meant for individuals whom they had identified in their own questionnaire as ‘important for managing their condition or disease’. The term ‘condition’ was used in questionnaires for high risk patients, and ‘disease’ in those for CVD patients. Patients were asked to give these questionnaires to their alters and provided with explanation on how to do this. Invitation letters for alters, providing concise information about the research, were enclosed to alter questionnaires along with informed consent forms. Patients and alters were provided with postal aid envelopes for returning their questionnaires. Data on clinical risk factors of patients at baseline of the RCT were gathered from patients’ medical records using the Epa Cardio abstraction tool [29], and were collected at the end of the RCT intervention program (performed from March 2014 until December 2014). This medical audit was performed by trained research assistants.

### Outcomes

Outcomes of this research were the description of network composition, patient health behavior, consisting of patient-reported physical activity, diet, and smoking, and the clinical indicators systolic blood pressure (SBP), low density lipoprotein cholesterol (LDL), and body mass index (BMI). Patient health behavior and the clinical indicators were dichotomous variables.

Health behaviors were measured using a composite questionnaire on: physical activity *(Rapid Assessment of Physical Activity* (RAPA), 9 items [30]); diet (*reduced Rapid Eating and Activity Assessment* (REAP-s), 12 items [31]); and smoking *(MID-SIZED Model*, 8 items [32]).

Physical activity was considered to be healthful if item 6 of the RAPA *(‘I do 30 minutes or more a day of moderate physical activities*, *5 or more days a week’)* **or** 7 *(‘I do 20 minutes or more a day of vigorous physical activities*, *3 or more days a week’)* was answered affirmatively. Diet was assessed with the REAP-s which asks to indicate how often (usually/often, sometimes, rarely/never, or not applicable) one engages in several unhealthful dietary habits in an average week. The REAP-s assesses habits on intake of grains, fruits and vegetables, calcium/dairy, saturated fat, and sugar. Patients who scored a maximum of two items ‘usually/often’ were considered to have healthful diet habits. Current smoking status was measured using one item from the Mid-sized Model. This item had four categories (‘yes, I smoke’, ‘no I quit smoking in the past 6 months’, ‘no, I quit smoking more than 6 months ago’, and ‘no, I never smoked’) which was recoded to a dichotomous variable (smoking vs non smoking).

Three clinical indicators of patients were abstracted from medical records using the Epa Cardio abstraction tool [29]: SBP, LDL, and BMI. Elevated SBP was defined as SBP > 140 mmHg. Elevated LDL was defined as LDL > 2.5 mmol/l. BMI was calculated by dividing patients’ weight by the square of height in meters. Overweight was scored with BMI > 25 kg/m2.

### Measures

#### Descriptive variables

Descriptive data of patients and their alters on age, sex, ethnicity, marital status, educational level, and working status were gathered using items from the Epa Cardio abstraction tool [29] in questionnaire booklets of the RCT for patients and in questionnaires for alters respectively.

#### Individual characteristics

Individual characteristics of patients were: patient activation *(Patient Activation Measure*, PAM [33]), therapy adherence *(Medication Adherence Measure* [34]*)*, and depressive symptoms *(Patient Health Questionnaire*, PHQ-9 [35]) and were measured using questionnaire booklets of the RCT. Higher total scores on these measures indicated higher patient activation, therapy adherence, and more depressive symptoms respectively. Alters completed the PHQ-9 as well, a cutoff score of 5 or higher indicated presence of depressive symptoms [35].

#### Alter health behavior

Health behavior of alters was measured using a composite questionnaire which was identical to that completed by patients (physical activity; RAPA [30], diet; REAP-s [31], smoking status; MIDSIZED MODEL [32]). Scoring rules for defining healthful physical activity, diet, and smoking status were also identical to those applied to patient data.

#### Social networks

Alters of patients were identified using two questions. First patients were asked to mention one person whom they considered to be most important for managing their condition or disease and health-related behaviors. It was explained that these included diet-, physical exercise-, and (if applicable) smoking habits. It was also stated that this person does not need to be ‘most important’ for a specific reason and does not need to be part of the patient’s personal environment. Second, patients were asked to name a maximum of three persons (other than their ‘most important other’) they considered important for managing their condition or disease. It was again explained that these persons did not need to be important for any specific reason. We emphasized that persons mentioned in this question did need to be part of patients’ personal environment. Patients were asked to appoint type of relation with each of their alters, response categories consisted of family, friends, acquaintances, or others.

### Data analysis

SPSS (version 22) was used for all analyses. All analyses were performed two tailed, using p < .05 indicating significance.

#### Construction of social network composition

We followed an identical procedure for the construction of all network compositions variables. First, we counted the number of alters with a certain behavior or trait who were present within patients’ networks. As few patients had more than one alter with a particular characteristic, we decided to dichotomize the social network composition variables. The resulting variables then, represented presence of one or more alters with a specific behavior or trait. Absence of alters with the behavior or trait of interest was used as reference categories. Note that data of patients without alters were not used in the construction of the following network components; presence of individuals with healthful behaviors, without depressive symptoms, and with specialized knowledge. This approach was chosen as presence of alters without a specific behavior or trait represented a different category than not having alters at all.

#### Presence of important others

Data from patients’ network questionnaires were used to create a dichotomous variable (important others present versus absent) using the items inferring on ‘your most important other’ and ‘name 3 others who you consider important for managing condition or disease’.

#### Presence of individuals with healthful behaviors

Four variables were created to test this network component using data from the RAPA (physical activity), REAP-s (diet), and a dichotomous item for smoking from alters’ questionnaires.

First, for assessing the influence of separate health behaviors of alters, three variables were created indicating whether an alter(s) with 1) healthful physical activity, 2) healthful diet habits, and 3) non-smoking habits, was present in patients’ networks.

Second, a dichotomous item for presence of alters with overall healthful behavior was created. Alters were considered to have overall healthful behavior when they engaged in healthful physical activity, *and* kept a healthful diet, *and* didn’t smoke.

#### Presence of individuals without depressive symptoms or depression

The PHQ-9 from alters questionnaires was used for assessment of depressive symptoms. Alters with a total score lower than 5 were scored as without depressive symptoms.

#### Presence of individuals with specialized knowledge on health, particularly health professionals

For assessing presence of persons with specialized knowledge on health in networks, data on occupation of alters were used using data from alter questionnaires. Alters with any job in health care were considered as having specialized knowledge on health.

#### Hypotheses testing

Logistic regression models were used for hypothesis testing, using General Equation Estimation (GEE) modeling to account for possible clustering due to sampling of patients from general practices. The working correlation matrix was specified as exchangeable and robust sandwich estimators were used.

A two step procedure was used to obtain parsimonious multivariate-adjusted models for testing of social network composition predictors. First, bivariate tests of the six social network composition factors and eleven patient predictors were performed. Second, predictors with p-values up to 0.10 were entered in multivariate-adjusted models. Six multivariate-adjusted models were specified, three models using patient health behavior (physical activity, diet, and smoking) as outcomes and three models using patient clinical risk factors (SBP, LDL, and BMI) as outcomes. Social network composition predictors consisted of the six variables specified in the hypotheses (presence of alters, presence of alters with healthful physical activity, healthful diet, non-smoking habits, and overall healthful behavior, alters without depressive symptoms, and alters with specialized knowledge on health). Patient predictors consisted of age, sex, education (high (completed higher vocational training or university) vs low education (vocational training or lower), marital status (relation (being married or having a partner) vs single), working status (employed vs unemployed), patient group (CVD vs high risk), RCT trial arm (intervention vs control), individual characteristics (patient activation and depressive symptoms), and health behaviors (physical activity, diet, and smoking, provisory on the dependent variable of the analysis). Originally, we planned to include nationality and primary language as patient control predictors. Almost all respondents had the Dutch nationality and language, so we decided to omit these variables from the analyses.

#### Alter participation

Additional analyses were performed to assess whether participation of alters was related to the six patient outcomes. Therefore, a dichotomous item was constructed, representing ´all or some alters participating´ vs ´no alters participating´, which was tested with logistic regression analyses using GEE modeling.

### Sensitivity analyses

#### Negative network composition

Three sensitivity analyses were performed. First, our hypotheses are phrased positively, so that patients with networks containing individuals with healthful behaviors will be more likely to engage in healthful behaviors themselves. However, if these hypotheses hold, an opposite pattern for negative network composition is just as likely to occur. Therefore, we tested the additional propositions that patients will be less likely to have healthful behaviors and favorable clinical risk factors, if their network contain: one or more alter(s) who hold unhealthful behaviors, alter(s) with depressive symptoms, and alter(s) without specialized knowledge on health. We followed a similar approach for construction of negative network composition predictors and for the specification of multivariate-adjusted models for testing these predictors as for the positively phrased predictors. The six multivariate-adjusted models were repeated with negative social network composition predictors using patient health behavior (physical activity, diet, and smoking) and risk factors (SBP, LDL, and BMI) as outcomes.

#### Mixed network composition

Second, network composition characteristics were tested using dichotomous items representing presence of one or more alters with certain behaviors versus absence of alters with these behaviors. As such, we assessed effects of presence of alters with either healthful or unhealthful behaviors. For assessing the effect of networks in which both alters with healthful and unhealthful behaviors or traits were present (“mixed network composition”), we created six ordinal variables for each of the characteristics of interest (alters’ physical activity, diet, smoking, overall health behavior, depression, and knowledge of health). Categories of these variables consisted of; 1) both alters with healthful and unhealthful behavior or trait present in networks, 2) alters with unhealthful behavior/trait present, 3) alters with healthful behavior or traits present. The last category was used as the reference category. These were tested bivariately using health behaviors and clinical risk factors as outcomes with logistic regression analyses using GEE modeling.

#### Psychological controls

Third, we explored the relative importance of psychological characteristics on outcomes in this study of network composition. Therefore, we examined the multivariate-adjusted regression models which included psychological variables and in which network components became non-significant. Effects of these network components were reconsidered by repeating these analyses while excluding the psychological variables.

## Results

### Response rates

A total of 1104 patients from 25 general practices, were invited to participate in this study. A total of 657 patients completed network questionnaires, an overall response rate of 60%. Alter response rate was considered in terms of network completeness. 477 patients reported to have one or more alters. Of 159 patients, all their alters participated in this study (33.3%), of 101 patients at least one but not all alters participated (21.2%), and of 217 patients none of their alters participated (45.5%).

### Sample & social networks characteristics

Table 1 provides descriptive data of patient characteristics and patients’ social networks. Patients had a mean age of 72.4 years, 32% was female, and 44% had established CVD. 73% of patients reported to have at least one alter. Data on type of relation were available for 382 alters, most (85%) were family of patients, 4% were friends, 2% were acquaintances, and 9% of relations were described as ‘other’.

**Table 1:** Descriptive data.

*Patient characteristics*		n (%) or mean (SD)	n
Age		72.44 (9.4)	657
Sex	Female	212 (32.3%)	657
Nationality	Dutch	622 (95.5%)	651
Primary language	Dutch	637 (98.9%)	644
Educational level	High	190 (29.4%)	646
Marital status	Relation	517 (79.4%)	651
Work	Employed	111 (17%)	653
Patient group	CVD	286 (43.5%)	657
TICD trial arm	intervention	384 (58%)	657
Patient activation	PAM total score	42.29 (6.67)	608
Therapy adherence	MMAS total score	1.28 (0.63)	127
Depressive symptoms	PHQ total score	2.41 (3.41)	646
Physical activity	Healthful*^1^	331 (52.4%)	632
Diet	Healthful	388 (60.3%)	643
Smoking	Yes	70 (10.8%)	646
Cholesterol	LDL>2.5 mmol/l	173 (71.2%)	243
Systolic blood pressure	SBP>140 mmHg	194 (53.6%)	362
Weight	BMI>25	119 (73%)	163
***Social network characteristics***		n (%)	n
Significant others	Present	477 (72.6%)	657
**Positive network composition**	***Presence of alters with/who*:**		
Healthful physical activity		170 (64.45%)	264
Healthful diet habits		219 (80.2%)	273
Non smoking		250 (91.2%)	274
Overall healthful behavior		119 (44.2%)	269
Without depressive symptoms		246 (91.4%)	269
With specialized knowledge on health		42 (30.9%)	136
**Negative network composition**	***Presence of alters with/who*:**		
Unhealthful physical activity		155 (58.7%)	264
Unhealthful diet habits		105 (38.5)	273
Smoking		48 (17.5)	274
Overall unhealthful behavior		198 (73.6)	269
With depressive symptoms		63 (23.4%)	269
Without specialized knowledge on health		108 (79.4%)	136
Mixed*^2^ **network composition**	***Presence of alters with/who*:**		
Physical activity		61 (23.1%)	264
Diet		51 (18.7%)	273
Smoking		24 (8.8%)	274
Overall health behavior		48 (17.8%)	269
Depressive symptoms		48 (17.8%)	269
Specialized knowledge on health		14 (10.3%)	136

*^1^ “healthful” was defined as follows, for physical activity: affirmative score on items 6 or 7 of the RAPA, for diet: a maximum score of two items on the REAPs as “usually/often”, for overall health behavior: engaging in health physical activity, diet and non-smoking,

*^2^Mixed networks contain both alters with healthful and alters with unhealthful behaviors or traits

Results for the impact of network composition on physical activity, diet, and smoking are presented in Table 2.

**Table 2:** Social network composition & patient health behaviors.

		PHYSICAL ACTIVITY			DIET				SMOKING		
		Bivariate		Multivariate	Bivariate		Multivariate		Bivariate		Multivariate
							*Model 1*	*Model 2*			
		OR (95%CI)	n	OR (95%CI)	OR (95%CI)	n	OR (95%CI)	OR (95%CI)	OR (95%CI)	n	OR (95%CI)
**POSITIVE NETWORK CHARACTERISTICS**											
***Presence of*:**											
Any alter	Yes	0.90 (0.65–1.24)	632		0.99 (0.65–1.48)	643			0.96 (0.62–1.49)		
	No										
Physically active alter(s)	Yes	1.02 (0.62–1.67)	255		1.51* (1.04–2.19)	259		1.26 (0.73–2.19)	1.31 (0.57–2.98)	261	
	No										
Alter(s) with healthful diet	Yes	***1*.*81*** ***(0*.*93–3*.*52)***	264	1.41 (0.63–3.18)	1.33 (0.70–2.51)	268			0.29** (0.12–0.69)	270	0.57 (0.19–1.69)
	No										
Non smoking alter(s)	Yes	2.84** (1.30–6.18)	265	2.05 (0.73–5.75)	2.75* (1.05–7.16)	269		2.82 (0.50–16.04)	0.08*** (0.02–0.27)	271	0.17** (0.05–0.60)
	No										
Alter(s) with overall healthful behavior	Yes	1.28 (0.79–2.06)	260		2.01*** (1.45–2.80)	264	2.14*** (1.52–3.02)		0.66 (0.28–1.52)	266	
	No										
Alter(s) without depressive symptoms	Yes	0.95 (0.38–2.35)	261		0.92 (0.45–1.89)	264			0.30* (0.12–0.75)	266	0.53 (0.14–2.02)
	No										
Alter(s) with specialized knowledge	Yes	0.70 (0.36–1.35)	132		***0*.*59*** ***(0*.*34–1*.*03)***	134		0.47* (0.24–0.89)	0.88 (0.32–2.42)	136	
	No										
**N in multivariate-adjusted model**				243			248	124			247

Multivariate-adjusted tests: network predictors were controlled for patient characteristics which were significantly related to the outcome of interest in bivariate testing. These were: for physical activity; sex, patient activation, depressive symptoms, diet, and smoking status. For diet; sex, patient activation, and smoking status. For smoking; age, education, working status, patient group, depressive symptoms, diet, and physical activity.

Estimated intercepts were omitted from the table.

*** = p < .001,

** = p < .01,

* = p < .05,

bold and cursive = p > .05 and < .10.

### Physical activity

#### Bivariate logistic GEE regressions

Two network components were related to healthful physical activity: presence of alters with a healthful diet (OR 1.81, 95%CI 0.93–3.52), and presence of non smoking alters (OR 2.84, 95%CI 1.30–6.18).

#### Multivariate-adjusted logistic GEE model

None of the network composition variables remained significant in the multivariate-adjusted model controlled for patient characteristics. These included sex (OR 1.49, 95%CI 0.90–2.47), patient activation (OR 1.05, 95%CI 1.00–1.10), depressive symptoms (OR 0.85, 95%CI 0.77–0.93), diet (OR 1.74, 95%CI 1.03–2.92), and smoking status (OR 0.54, 95%CI 0.23–1.28).

### Diet

#### Bivariate logistic GEE regressions

Results of bivariate analyses showed that four network components were related to healthful patient diet: presence of alters with healthful physical activity (OR 1.51, 95%CI 1.04–2.19), presence of non smoking alters (OR 2.75, 95%CI 1.05–7.16), presence of alters with overall healthful behavior (OR 2.01, 95%CI 1.45–2.80), and alters with specialized knowledge (OR 0.59, 95%CI 0.34–1.03).

#### Multivariate-adjusted logistic GEE models

Two multivariate-adjusted models were estimated; one including the network component ‘presence of alters with overall healthful behavior’ and one including the variables ‘presence of physically active alters’ and ‘presence of non smoking alters’ along with ‘presence of alters capable of providing information’.

Odds for healthful diet, relative to an unhealthful diet, were 114% higher for patients with networks that contained alters with overall healthful behavior (OR 2.14, 95%CI 1.52–3.02) compared to patients whose networks did not contain such alters. Effects of presence of physically active alters and of non smoking alters reduced to non significance in the multivariate-adjusted model whereas the effect of presence of alters with specialized knowledge became significant (OR 0.47, 95%CI 0.24–0.89). In other words, the odds for healthful diet were 53% lower for patients whose networks contained alters capable of providing information on CVRM.

In the multivariate-adjusted models we controlled for the following patient characteristics: sex (Model 1 OR 0.32, 95%CI 0.17–0.60, Model 2 OR 0.33, 95%CI 0.15–0.75), patient activation (Model 1 OR 1.01, 95%CI 0.96–1.06 and Model 2 OR 1.01, 95%CI 0.96–1.07), and smoking status (Model 1 OR 0.50, 95%CI 0.23–1.08, Model 2 OR 0.54, 95%CI 0.19–1.51).

### Smoking

#### Bivariate logistic GEE regressions

Three network components were related to patient smoking: presence of alters with a healthful diet (OR 0.29, 95%CI 0.12–0.69), presence of non-smoking alters (OR 0.08, 95%CI 0.02–0.27), and presence of alters without depressive symptoms (OR 0.30, 95%CI 0.12–0.75).

#### Multivariate-adjusted logistic GEE model

One network component remained significant in the multivariate-adjusted model of patient smoking; odds for smoking were 83% lower for patients whose social networks contained non-smoking alters (OR 0.17, 95%CI 0.05–0.60). The model was controlled for the following patient characteristics: age (OR 0.97, 95%CI 0.94–1.01), education (high vs low, OR 0.70, 95%CI 0.28–1.77), working status (employed vs unemployed, OR 2.10, 95%CI 0.70–6.32), patient group (CVD vs high risk, OR 0.80, 95%CI 0.30–2.17), depressive symptoms (OR 1.06, 95%CI 0.95–1.18), diet (OR 0.54, 95%CI 0.22–1.31), and physical activity (OR 0.47, 95%CI 0.23–0.99)

### Clinical risk factors

None of the social network components were related to any of the clinical indicators (SBP, LDL, and BMI) in the bivariate analyses and therefore were not tested in multivariate-adjusted models. Bivariate estimates for effects of social network components and of patient characteristics are included in Appendix A in S4 File.

### Alter participation

Alter participation (all or some alters participating vs none of the alters participating) was not related to any of the outcomes.

### Sensitivity analyses

#### Negative social network composition & patient health behavior

Results for physical activity, diet, and smoking are presented in Table 3. Overall, results mirrored those of positive network composition; relations had opposite directions for negative network components. Results from multivariate-adjusted models showed that having alter(s) without specialized knowledge was related to increased odds for healthful physical activity (OR 3.48, 95%CI 1.21–10.10), having alters with overall unhealthful behavior was related to reduced odds for healthful patient diet (OR 0.48, 95%CI 0.30–0.75), and having smoking alter(s) was related to increased odds for patient smoking (OR 5.53, 95%CI 2.11–14.52).

**Table 3:** Negative social network composition & patient health behaviors.

		PHYSICAL ACTIVITY			DIET				SMOKING		
		Bivariate		Multivariate	Bivariate		Multivariate		Bivariate		Multivariate
							*Model 1*	*Model 2*			
		OR (95%CI)	n	OR (95%CI)	OR (95%CI)	n	OR (95%CI)	OR (95%CI)	OR (95%CI)	n	OR (95%CI)
**POSITIVE NETWORK CHARACTERISTICS**											
***Presence of*:**											
Physically inactive alter(s)	Yes	0.76 (0.45–1.27)	255		0.77 (0.52–1.14)	259			0.58 (0.29–1.17)	261	
	No										
Alter(s) with unhealthful diet	Yes	0.93 (0.54–1.61)	264		***0*.*61*** ***(0*.*36–1*.*04)***	268		0.63 (0.35–1.14)	2.34** (1.26–4.33)	270	1.25 (0.65–2.51)
	No										
Smoking alter(s)	Yes	0.59* (0.37–0.96)	265	0.86 (0.37–2.03)	0.57* (0.33–0.98)	269		0.78 (0.37–1.64)	6.79*** (2.58–17.86)	271	5.53** (2.11–14.48)
	No										
Alter(s) with overall unhealthful behavior	Yes	0.63 (0.32–1.22)	260		0.48** (0.30–0.77)	264	0.48** (0.30–0.75)		1.90 (0.87–4.17)	266	
	No										
Alter(s) with depressive symptoms	Yes	1.05 (0.59–1.85)	261		0.73 (0.44–1.23)	264			1.43 (0.67–3.03)	266	
	No										
Alter(s) without specialized knowledge	Yes	***2*.*44*** ***(0*.*93–6*.*43)***	132	3.48* (1.21–10.10)	1.02 (0.40–2.63)	134			0.83 (0.24–2.86)	136	
	No										
**N in multivariate-adjusted model**				121			248	251			252

Multivariate-adjusted tests: network predictors were controlled for patient characteristics which were significantly related to the outcome of interest in bivariate testing. These were: for physical activity; sex, patient activation, depressive symptoms, diet, and smoking status. For diet; sex, patient activation, and smoking status. For smoking; age, education, working status, patient group, depressive symptoms, diet, and physical activity.

Estimated intercepts were omitted from the table.

*** = p < .001,

** = p < .01,

* = p < .05,

bold and cursive = p > .05 and < .10.

#### Negative social network composition & clinical risk factors

Results for SBP, LDL, and BMI are included in Appendix B in S4 File. One network component was related to one patient health outcome; odds for elevated SBP were increased for patients whose networks contained alters with unhealthful diets (OR 2.21, 95%CI 1.16–4.21). This effect remained significant (OR 2.17, 95%CI 1.11–4.28)) controlled for age (OR 1.06, 95%CI 1.02–1.11), work status (OR 1.54, 95%CI 0.66–3.62), and patient group (OR 0.72, 95%CI 0.40–1.28).

#### Mixed social network compositions

Mixed network composition for alters’ health knowledge was related to increased odds for healthful patient physical activity (OR 3.92, 95%CI 1.24–12.43), and mixed network composition for alter diet was related to elevated SBP of patients (OR 2.73, 95%CI 1.15–6.51). None of the other mixed social network components were related to any of the outcomes.

#### Psychological variables

Multivariate-adjusted models were repeated for positive and negative network composition while excluding the psychological control variables patient activation and depressive symptoms. Effects of network composition on patient’ physical activity, diet, and smoking did not change when these psychological variables were excluded from the models.

## Discussion

In this observational study we explored linkages between vascular patients’ network composition on the one hand and health behaviors and clinical health indicators on the other hand. Controlling for demographic, personal, and psychological characteristics, we found a few linkages: alters’ smoking behavior was related to patients’ smoking and alters’ overall health behavior was related to patients’ diet. None of the hypothesized network components were related to clinical indicators, except that the presence of alters with unhealthful diet habits in patients’ networks was related to increased odds for elevated SBP of patients. Overall, these findings only partly support the notion that health-related behaviors are associated with patients’ social network composition.

Our result that odds for smoking were increased in networks which contain other smokers is in line with several prior studies [5,9,36], which showed that smoking as well as other behaviors appeared to be social contagious or social transmissible [4–8], as often indicated by clustering of particular behaviors in networks. Several processes have been proposed to explain clustering. First, clustering may occur because of homophily: the selection of contacts who have similar traits or behaviors. Second, behaviors of one person trigger similar behaviors in another, a process termed induction. Third, similar experienced external causes may cause individuals to share traits or behaviors [37]. The observational design of our study does not allow to infer which mechanism is responsible for the identified relation between smoking of patients and their alters. However, in the study population of middle aged and older people, with relatively stable social networks, it may reflect mutual reinforcement of smoking behaviors rather than selection of smoking network members.

Furthermore, we found that patients’ odds for healthful diet were increased if their networks contained alters with overall healthful behavior. We are unaware of previous research on overall health behavior of alters and specific components of patient health behavior. Possibly this result may indicate the presence of another underlying network mechanism than clustering. Prior research suggested that social contacts can be beneficial for spread of information and role modeling [38], of which alters with overall healthful behavior can be likely candidates. Other studies noted that social contacts may provide encouragement [39]. Being able to master healthful behaviors themselves, such alters may likely be persons to encourage patients to achieve particular health behaviors.

No associations between alters’ and patients’ physical activity and diet respectively, were found, which is dissimilar to results of studies on contagion of several health related behaviors [40]. There are several potential explanations. First, due to the low response rate of alters, sample size and power in the multivariate-adjusted analyses was limited. Also, network effects were controlled for several patients characteristics. Among these were characteristics which were hypothesized to be influenced by networks themselves as well (e.g. analyses for physical activity were controlled for depressive symptoms, diet, and smoking behaviors). Longitudinal research is needed to unravel such relations. Also, control variables may have had possible mediating roles (e.g. depressive symptoms). As such, it is possible that by using our modeling approach we overadjusted effects of network composition. Second, we may have applied a too broad definition of a support network. Although support networks have been shown to positively influence health [41], other studies showed that specific persons may be important for influencing health behavior. Previous research on older adults found homophily for health behaviors in close contacts, or in the ‘inner circle’ of networks [36]. Other research showed that, when it comes to influencing behavior, not all persons are of equal importance and that particular connections may be more likely to exert influences [42,43]. Furthermore, research indicated the importance of the spouse for several health behaviors, cognitions, and health outcomes [43,44]. If influences on diet and physical activity are dependent on specific persons from networks, our network definition may have led us to include persons in our analyses which are not close enough to substantially affect patient behavior, thereby distorting effects of persons who may have had considerable influences. This may be especially true for diet in the context of the study population which tend to mention family as members of their support network and the fact that eating tends to take place with family members. Considering the older age of patients, they are likely to eat most often with their spouse who may have therefore have had more influence on patient diet than other family members and other persons. Third, it is possible that patient characteristics are more important for understanding diet and physical activity than network composition. Some support for this thought can be found considering the several bivariate effects of network components which did not remain significant when controlled for patient factors and the results on clinical risk factors, with several patient factors significant in the multivariate-adjusted models and only one network component contributing to clinical indicators.

Effects of having alters with specialized knowledge on health (social capital) were contrary to our hypotheses for some outcomes and lacking for other outcomes. This is dissimilar to other research [23]. We may have found different results because of our specific definition of social capital as having persons (that is health professionals) in networks which specialized knowledge on health. Two main interpretations then may explain why we have found different results. First, participants in this study may have been not in need of information on CVRM as currently available information sources on health care (in the Netherlands) are wide ranging, with many on the Internet. Also, although having health professionals within ones network implies having access to reliable information, it remains uncertain whether patients also received this information. Previous research on informational support indicated information works best when it is needed, the so called Matching hypothesis [41]. ‘Mismatching’ then, may occur when health professionals are present but when information is provided at wrongly timed occasions, and perhaps too often. Mismatching may then result in negative interactions, which may be particularly relevant in this study given our low response rate of alters. It may be that especially alters who felt committed to patients and their health participated. Such engaged alters may become over involved, which may put strain on relations. This notion may be supported by the one effect found for social capital which was related to reduced odds for appropriate diet. Second, although we hypothesized patients to benefit when alters with specialized knowledge were present in networks, it may be also be possible that the effect runs the opposite way. People with unhealthy behaviors may be more likely to require help from professionals with specialized knowledge. Our negative effect may then reflect patients in need of information (i.e. those with unhealthful behaviors), seeking out or contacting alters who can provide these.

We found virtually no effects of network composition on clinical risk factors. A plausible route for influences of network components is by first influencing health behavior of patients, which then result in particular outcomes of clinical risk factors. Given that only a few of our hypothesized network components were related to patient health behavior, it is then not surprising that clinical risk factors were unaffected by network composition.

Behavioral and clinical outcomes were overall not different for patients with and without a support network (i.e. presence of alters). This is in contrast to research on social isolation in patients with chronic conditions [43]. However, in this research we constructed networks on persons which were considered important for managing disease. Patients without such alters do not necessarily have to be socially isolated, and may have well had other (type of) contacts with possible influences on health behavior and outcomes. Our result also seems in contrast to research on social support, which has mainly reported positive influences of support on health [14]. However, in line with our finding, several other studies reported no effects of support as well [45,46] or identified negative effects of networks [47]. Other studies noted that, in addition to social support, other mechanisms such as social influence and social engagement are important for understanding the role of the social environment in influencing health as well [12]. Support networks identified in this study mainly consisted of family of patients. A previous study on older adults attempting to identify dimensions of support networks, showed that family was not associated with health outcomes while social engagement was significantly related to both psychological and physical health outcomes [46]. Another study differentiating types of people within older adults networks found that only contacts with people with whom socializing was enjoyed were related to self rated-health [47]. Such results may emphasize the relevance of other, or more specifically defined, social mechanisms than social support. Additionally, other studies showed that particular connections within networks are of more importance than others [42–44]. In our study, we may have not been able to sufficiently tap into such mechanisms using our definition of support networks, or we may have not identified, or differentiated between, specific contacts with particular importance. It is also possible that social support by family members did not have pronounced effects in this population of vascular patients, or that positive and negative network influences cancelled each other out.

Other research found stronger evidence of the protective effects of social networks on health than this study. However, these previous studies considered whole networks instead of personal support networks, other network characteristics, including social integration [48], social connectedness [49]), other type of networks (e.g. friendship [50]), and other structural characteristics of networks (e.g. network size [51] or diversity [43,52]). Our study may suggest that other network characteristics or wider social structures can be of more importance for behavior and clinical risk factors than the presence of alters with certain features in an individual support network. Future research should focus on the identification of these characteristics or structures.

Previous network research gave less attention to psychological characteristics as determinants of health behaviors. In this study, network influences were controlled for patient activation and depressive symptoms as psychological constructs. Our multivariate-adjusted analyses showed that these variables indeed were associated with physical activity and LDL. It should also be noted that the prevalence of depressive symptoms in our sample was rather low for both patients and their alters. Our results indicate that to enhance understanding of health behavior and clinical risk factors, and the relative importance of individual and social influences in health, future research should take both into account.

Strengths of the study included the use of validated measures of health behavior, the use of clinical indicators abstracted from medical records, the adjustment for psychological factors in the regression models, and the inclusion of patients’ alters in the study. Limitations of this research include the following. First, the observational design does not allow for causal inferences between network composition and health behaviors and clinical risk factors. As such, the results of this study should be interpreted carefully and future research is needed to establish causal relations between network influences and health outcomes. Second, the response rate of alters was low and we cannot exclude the possibility of a selection bias within this group. Therefore, care is warranted for the interpretation and generalization of our results. Also, and although the response of patients was reasonable, the low response of alters left us with a limited sample size and reduced power in the regression models. Third, our patient sample may have been prone to selection bias as well. It may be that patients having merely positive contacts were more likely to participate than patients whose network comprised more negative contacts, or then patients without a network.

Furthermore, we excluded patients with diabetes, which represent a group with high risk for CVD. This study was tied to the sampling strategy of a RCT aiming to improve primary care for CVRM. As primary care for diabetes has received much attention (supportive materials, continuing education programs, additional reimbursement) in the Netherlands, inclusion of patients with diabetes would have compromised outcomes of the RCT. Therefore, care is warranted for generalizing our results to other patient groups. Fourth, we tested our hypotheses in six regression models. Such repeated testing increases risk for type 1 error rate, for which solutions such as adjustment of p-values are available. However, we decided not to adjust the threshold for statistical significance given the explorative aim of the study, and because such adjustments come with the risk of enhanced type II error rate, which can be especially relevant given our smaller sample size. Fifth, several aspects of our specific approach may have limited the ability to detect associations because of limited variability. These include the relatively small number of alters we were able to include in the study. Related, we cannot be sure that more alters would have been identified if we had employed another (less broad) definition of networks. Also, in multivariate-adjusted tests of network composition, we included each patient characteristic that was bivariately related to the outcome of interest. Among these were characteristics which may be mediating variables (e.g. depression). Also, in analyses on health behavior outcomes, we adjusted for other health behaviors (e.g. in analyses on smoking, we adjusted for diet and physical activity). This approach, and the limited sample size, may have led to overadjustment of potential effects of network composition.

Sixth, questionnaires to measure networks were not validated. Seventh, an interval up to a maximum of three months between completing RCT questionnaires and sending network questionnaires was needed. It remains unsure whether and how this affected results. A too short interval between receiving both questionnaires may be discouraging to participate, whereas a too large interval may have caused a loss of interest or motivation to participate.

### Conclusions

In this explorative study, we found some evidence for influences of network composition on patients’ health behavior. Odds for patient smoking were reduced if their networks contained non-smoking alters and increased odds for healthful patient diet habits were found if their networks contained alters with overall healthful behavior. We included alters of patients and controlled effects of network composition for several psychological variables, which are known to influence several patient health behaviors by themselves. Several identified effects of network components reduced to non-significance when controlled for such psychological characteristics of patients.

As such, this study indicated it is important to take network composition into account but also that other influences matter as well. Future research is warranted to further examine relations between network composition and health outcomes. It may be noted that several aspects of our study may have influenced our results and should be taken into account in future studies as well. These include that the modeling of predictors of outcomes could have taken an alternative approach, in which mediating or moderation roles of individual and psychological characteristics of patients are explicitly modeled. In addition, the inclusion of alters of patients needs further attention. Factors to be considered include the identification of alters as well as the enrollment of these individuals in the study.

## Supporting information

S1 File Waiver ethical approval (original version in Dutch). (DOCX)

S2 File Waiver ethical approval (translated). (DOCX)

S3 File Social networks questionnaire. (TIF)

S4 File Appendix. (DOCX)

## Abbreviations

BMI: body mass index
CVD: cardiovascular disease
CVRM: cardiovascular risk management
GEE: generalized estimating equations
ICPC: International classifications of primary care
LDL: low density lipoprotein cholesterol
RCT: randomized controlled trial, the larger RCT in which the network study was embedded
SBP: systolic blood pressure
