# Supplementary material for: Social network composition of vascular patients and its associations with health behavior and clinical risk factors
Source: PLoS One. 2017 Sep 28;12(9):e0185341. doi: 10.1371/journal.pone.0185341 (PMC5619748; doi:10.1371/journal.pone.0185341)
Supplement: S1 File — (DOCX) [file pone.0185341.s001.docx]

**Titel: Information sharing networks of health care providers and patients in cardiovascular risk management. A study in the “Tailored Implementation for Chronic Diseases’ (TICD) project**

**Registratienummer: 2013/298**

Geachte mevrouw Heijmans,

In antwoord op uw e-mail van 24 juni 2013 bericht ik u namens de CMO als volgt.

Bij uw e-mail heeft de CMO de volgende stukken ontvangen:

- Protocol, versie 24-06-2013

- Vragen voor in vragenlijstboek interventiestudie, groep 1, versie 24-06-2013, totaal vragen = 3

- Vragen voor in vragenlijstboek interventiestudie, groep 2, versie 24-06-2013, totaal vragen = 3

- Vragenlijst voor patiënten met HVZ, versie 24-06-2013, deze vragenlijst bevat in totaal 12 vragen (subvragen zijn meegeteld)

- Vragenlijst voor patiënt met verhoogd risico op HVZ, versie 24-06-2013, deze vragenlijst bevat in totaal 12 vragen (subvragen zijn meegeteld)

- Vragenlijst voor alters van patiënten, versie 24-06-2013, deze vragenlijst bevat in totaal 47 vragen

- Begeleidende brief voor telefonische afname patiënten, versie 24-06-2013

- Begeleidende brief vragenlijst patiënten, versie 24-06-2013

- Uitnodigingsbrief voor alters van patiënten, versie 24-06-2013

- Vragenlijst voor zorgverleners, versie 24-06-2013

- Vragenlijst voor alters van zorgverleners, versie 24-06-2013

- Begeleidende brief voor zorgverleners, versie 24-06-2013

- Uitnodigingsbrief voor alters van zorgverleners, versie 24-06-2013

Met inachtneming van hetgeen in de Wet medisch-wetenschappelijk onderzoek (WMO) is bepaald is de CMO van oordeel dat het onderzoek niet onder de reikwijdte van WMO valt. Hieruit volgt dat voor de uitvoering van het onderzoek geen oordeel van de CMO als erkende commissie of van een andere erkende commissie is vereist.

In uw onderzoek wordt geen (nader) gebruik gemaakt van lichaamsmateriaal. Dit betekent dat voor de uitvoering van het onderzoek op grond van het ziekenhuisvoorschrift nader gebruik lichaamsmateriaal ook geen goedkeuring vereist is van de CMO als lokale toetsingscommissie.

Ik vertrouw erop u met deze e-mail van dienst te zijn.

Met vriendelijke groet,

Dr F. Huysmans, voorzitter

**Universitair Medisch Centrum St Radboud**

**Concernstaf Kwaliteit en Veiligheid - Commissie Mensgebonden Onderzoek**

Huispost 547, route 553

Postbus 9101

6500 HB Nijmegen

Telefoon: (024) 36 13154

E-mail: [cmo@iwkv.umcn.nl](mailto:cmo@medzaken.umcn.nl)

[http://portal.umcn.nl/organisatie/iwkv](http://portal.umcn.nl/organisatie/IWKV/Pages/home.aspx)

De inhoud van dit bericht wordt niet bevestigd middels een brief.
