# Supplementary material for: Social network composition of vascular patients and its associations with health behavior and clinical risk factors
Source: PLoS One. 2017 Sep 28;12(9):e0185341. doi: 10.1371/journal.pone.0185341 (PMC5619748; doi:10.1371/journal.pone.0185341)
Supplement: S2 File — (DOCX) [file pone.0185341.s002.docx]

**Title: Information sharing networks of health care providers and patients in cardiovascular risk management. A study in the “Tailored Implementation for Chronic Diseases’ (TICD) project**

**Registration number: 2013/298**

Dear ms. Heijmans,

In reply at your e-mail (dated June 24 2013) I notice you on behalf of the CMO as follows.

In your e-mail the CMO has received the following pieces:

- Protocol, version 24-06-2013

- Questions to be included in the questionnaire booklet of the intervention study Group 1, version 24-06-2013, total number of questions = 3

- Questions to be included in the questionnaire booklet of the intervention study Group 2, version 24-06-2013, total number of questions = 3

- Questionnaire for patients with CVD, version 24-06-2013, this questionnaire contains a total of 12 questions (sub questions counted as well)

- Questionnaire for patients at high risk for CVD, version 24-06-2013, this questionnaire contains a total of 12 questions (sub questions counted as well)

- Questionnaire for alters of patients, version 24-06-2013, this questionnaire contains a total of 47 questions

- Accompanying letter for telephonic administration patients, version 24-06-2013

- Accompanying letter for questionnaire administration patients, version 24-06-2013

- Invitational letter for alters of patients, version 24-06-2013

- Questionnaire for health care professionals, version 24-06-2013

- Questionnaire for alters of health care professionals, version 24-06-2013

- Accompanying letter for health care professionals, version 24-06-2013

- Invitational letter for alters of health care professionals, version 24-06-2013

Considering what has been determined in the Law Medical-Scientific research (*in Dutch: WMO*), the CMO has determined that this law does not apply to this research. From this it follows that for the performance of this research no judgment of the CMO as accredited commission, or from another accredited commission, is required.

Your research does not consider using body materials. Therefore, based on the hospital prescription on further use of body materials, for the performance of the research no approval is needed from the CMO as local review committee as well.

I trust to be of service with this e-mail.

Kind regards,

Dr F. Huysmans, chairman

**Universitair Medisch Centrum St Radboud**

**Concernstaf Kwaliteit en Veiligheid - Commissie Mensgebonden Onderzoek**

Huispost 547, route 553

Postbus 9101

6500 HB Nijmegen

Telefoon: (024) 36 13154

E-mail: [cmo@iwkv.umcn.nl](mailto:cmo@medzaken.umcn.nl)

[http://portal.umcn.nl/organisatie/iwkv](http://portal.umcn.nl/organisatie/IWKV/Pages/home.aspx)

The content of this message is not confirmed by a letter.
