# Supplementary material for: Social network composition of vascular patients and its associations with health behavior and clinical risk factors
Source: PLoS One. 2017 Sep 28;12(9):e0185341. doi: 10.1371/journal.pone.0185341 (PMC5619748; doi:10.1371/journal.pone.0185341)
Supplement: S4 File — (DOCX) [file pone.0185341.s004.docx]

**S4 Appendix**

**Appendix A**

**Bivariate estimates on clinical risk factors**

|  |  |  | **SBP** | | | | | **LDL** | | | | | **BMI** | | | | |
| --- | --- | --- | --- | --- | --- | --- | --- | --- | --- | --- | --- | --- | --- | --- | --- | --- | --- |
|  |  |  | **OR** | **95%CI** | | | **n** | **OR** | **95%CI** | | | **n** | **OR** | **95%CI** | | | **n** |
| ***PATIENT CHARACTERISTICS*** | |  |  |  |  |  |  |  |  |  |  |  |  |  |  |  |  |
|  |  | Age | **1.06***** | **1.03** |  | **1.08** | 362 | 0.98 | 0.94 |  | 1.02 | 243 | 1.00 | 0.96 |  | 1.05 | 163 |
| Sex | | Female | 1.09 | 0.70 |  | 1.71 | 362 | 0.84 | 0.53 |  | 1.33 | 243 | 1.31 | 0.57 |  | 3.04 | 163 |
|  |  | Male |  |  |  |  |  |  |  |  |  |  |  |  |  |  |  |
| Education | | High | 1.06 | 0.71 |  | 1.58 | 356 | 1.57 | 0.85 |  | 2.89 | 242 | 0.73 | 0.34 |  | 1.58 | 161 |
|  |  | Low |  |  |  |  |  |  |  |  |  |  |  |  |  |  |  |
| Marital status | | Relation | 1.07 | 0.66 |  | 1.75 | 358 | **2.01*** | **1.04** |  | **3.91** | 243 | 1.13 | 0.59 |  | 2.16 | 162 |
|  |  | Single |  |  |  |  |  |  |  |  |  |  |  |  |  |  |  |
| Working status | | Employed | **0.44**** | **0.26** |  | **0.74** | 360 | 1.66 | 0.77 |  | 3.6 | 243 | 1.40 | 0.48 |  | 4.10 | 163 |
|  |  | Unemployed | |  |  |  |  |  |  |  |  |  |  |  |  |  |  |
| Patient group | | CVD | **0.65**** | **0.46** |  | **0.92** | 362 | **0.38*** | **0.21** |  | **0.71** | 243 | 1.13 | 0.58 |  | 2.20 | 163 |
|  |  | High risk |  |  |  |  |  |  |  |  |  |  |  |  |  |  |  |
| Trial arm | | Intervention | 1.20 | 0.70 |  | 2.05 | 362 | 0.67 | 0.39 |  | 1.14 | 243 | 0.74 | 0.35 |  | 1.58 | 163 |
|  |  | control |  |  |  |  |  |  |  |  |  |  |  |  |  |  |  |
| Patient activation* | | PAM | 1.00 | 0.96 |  | 1.04 | 337 | ***1.04*** | 0.99 |  | 1.09 | 228 | 1.00 | 0.96 |  | 1.05 | 157 |
| Medication adherence* | | MMAS | 0.95 | 0.68 |  | 1.34 | 325 | 1.18 | 0.81 |  | 1.72 | 226 | 0.82 | 0.37 |  | 1.81 | 151 |
| Depressive symptoms* | | PHQ | 1.02 | 0.97 |  | 1.08 | 355 | 1.07 | 0.98 |  | 1.17 | 241 | 0.94 | 0.87 |  | 1.01 | 162 |
| Diet | | Healthful | 0.81 | 0.61 |  | 1.07 | 354 | 1.33 | 0.80 |  | 2.22 | 241 | 0.66 | 0.32 |  | 1.34 | 162 |
|  |  | Unhealthful |  |  |  |  |  |  |  |  |  |  |  |  |  |  |  |
| Physical activity | | Healthful | 0.94 | 0.66 |  | 1.34 | 348 | 1.07 | 0.63 |  | 1.81 | 233 | ***1.52*** | 0.95 |  | 2.44 | 157 |
|  |  | Unhealthful |  |  |  |  |  |  |  |  |  |  |  |  |  |  |  |
| Smoking status | | Yes | 0.70 | 0.37 |  | 1.32 | 353 | 0.66 | 0.29 |  | 1.49 | 239 | 0.59 | 0.29 |  | 1.21 | 156 |
|  |  | no |  |  |  |  |  |  |  |  |  |  |  |  |  |  |  |
| ***NETWORK COMPOSITION*** | |  |  |  |  |  |  |  |  |  |  |  |  |  |  |  |  |
| ***Presence of:*** |  |  |  |  |  |  |  |  |  |  |  |  |  |  |  |  |  |
| Any alter | | Yes | 0.95 | 0.64 |  | 1.41 | 362 | 1.85 | 0.87 |  | 3.92 | 243 | 1.04 | 0.42 |  | 2.61 | 163 |
|  |  | No |  |  |  |  |  |  |  |  |  |  |  |  |  |  |  |
| Physically active alter(s) | | Yes | 1.32 | 0.62 |  | 2.80 | 150 | 0.83 | 0.36 |  | 1.93 | 100 | 0.70 | 0.18 |  | 2.70 | 71 |
|  |  | No |  |  |  |  |  |  |  |  |  |  |  |  |  |  |  |
| Alter(s) with healthful diet | | Yes | 0.73 | 0.31 |  | 1.74 | 156 | 1.36 | 0.36 |  | 5.16 | 103 | 0.58 | 0.15 |  | 2.22 | 74 |
|  |  | No |  |  |  |  |  |  |  |  |  |  |  |  |  |  |  |
| Non smoking alter(s) | | Yes | 0.71 | 0.24 |  | 2.06 | 157 | 0.82 | 0.20 |  | 3.30 | 105 | 2.85 | 0.67 |  | 12.07 | 74 |
|  |  | No |  |  |  |  |  |  |  |  |  |  |  |  |  |  |  |
| Alter(s) with | | Yes | 1.01 | 0.56 |  | 1.81 | 154 | 0.93 | 0.44 |  | 1.97 | 103 | 0.80 | 0.26 |  | 2.53 | 73 |
| overall healthful behavior | | No |  |  |  |  |  |  |  |  |  |  |  |  |  |  |  |
| Alter(s) without depressive | | Yes | 0.63 | 0.20 |  | 1.94 | 155 | 0.38 | 0.05 |  | 2.96 | 102 | 0.64 | 0.16 |  | 2.54 | 74 |
| symptoms |  | No |  |  |  |  |  |  |  |  |  |  |  |  |  |  |  |
| Alter(s) with specialized | | Yes | 0.72 | 0.30 |  | 1.75 | 75 | 1.10 | 0.34 |  | 3.63 | 53 | 1.00 | 0.30 |  | 3.37 | 35 |
| knowledge |  | No |  |  |  |  |  |  |  |  |  |  |  |  |  |  |  |

* total scores of the PAM, MMAS, and PHQ-9 were entered in analyses to assess influences of patient activation, medication adherence, and depressive symptoms respectively.

**Appendix B**

**Bivariate estimates of negative social network composition and clinical risk factors**

|  |  |  |  |  |  |  |  |  |  |  |  |  |  |  |  |
| --- | --- | --- | --- | --- | --- | --- | --- | --- | --- | --- | --- | --- | --- | --- | --- |
|  | **SBP** | | | | | **LDL** | | | | | **BMI** | |  |  |  |
|  | **Bivariate** | | | | | **Bivariate** | | | | | **Bivariate** | |  |  |  |
|  | **OR** | **95%CI** | | | n | **OR** | **95%CI** | | | n | **OR** | **95%CI** | | | n |
| Physically inactive alter(s) | 1.09 | 0.60 |  | 2.03 | 150 | 1.11 | 0.53 |  | 2.37 | 100 | 1.61 | 0.66 |  | 3.93 | 71 |
|  |  |  |  |  |  |  |  |  |  |  |  |  |  |  |  |
| Alter(s) with unhealthful diet | **2.21*** | **1.16** |  | **4.21** | **156** | 1.19 | 0.50 |  | 2.85 | 103 | 1.56 | 0.41 |  | 5.91 | 74 |
|  |  |  |  |  |  |  |  |  |  |  |  |  |  |  |  |
| Smoking alter(s) | 0.82 | 0.36 |  | 1.87 | 157 | 1.54 | 0.37 |  | 6.37 | 105 | 0.32 | 0.07 |  | 1.50 | 74 |
|  |  |  |  |  |  |  |  |  |  |  |  |  |  |  |  |
| Alter(s) with | 1.07 | 0.52 |  | 2.17 | 154 | 1.29 | 0.58 |  | 2.83 | 103 | 1.81 | 0.62 |  | 5.30 | 73 |
| overall unhealthful behavior |  |  |  |  |  |  |  |  |  |  |  |  |  |  |  |
| Alter(s) with depressive | 1.24 | 0.62 |  | 2.50 | 155 | 1.55 | 0.46 |  | 5.23 | 102 | 1.97 | 0.65 |  | 5.97 | 74 |
| symptoms |  |  |  |  |  |  |  |  |  |  |  |  |  |  |  |
| Alter(s) without specialized | 1.37 | 0.51 |  | 3.17 | 75 | 0.87 | 0.15 |  | 4.96 | 53 | 1.00 | 0.12 |  | 8.12 | 35 |
| knowledge |  |  |  |  |  |  |  |  |  |  |  |  |  |  |  |

**Appendix C**

**Deviations from the study protocol**

This study has an associated study protocol [26], of which we needed to make some deviations in the statistical analyses.

First, hypotheses were tested in a different form than stated in the protocol. Instead of testing that ‘a high number of alters’ with certain behaviors were related to patient’ health behavior and health status, we tested the influence of ‘the presence of alters’ with certain behaviors. ‘A high number of alters’ was originally formulated as the count of the number of alters with certain behaviors. As few alters had more than one alter with behaviors of interest, variables became too skewed to be entered into the analyses. Therefore, variables were dichotomized, contrasting having one or more alters with specific behaviors versus not having alters with specific behaviors in patients’ social networks. Concomitant with the alternative construction of network variables a shift in phrasing of hypotheses to ‘presence of alters with certain behaviors’ was made.

Second, we did not test the hypothesis that ‘alters with particular behaviors who are also connected among each other’ would be positively related to patient’ health behaviors and health status. Interconnectedness of alters was measured. However, data showed that almost all alters were connected among each other, thus leaving this variable with too little variation to be used in the analyses. Considering that most reported alters were family of patients, it is not surprising that interconnectedness was high.

Third, in the study protocol we noted that we would use risk score as outcome for high risk patients. This outcome was omitted from the research as we were unable to collect sufficient data for calculating risk scores. Instead of risk score, body mass index was used as outcome for both high risk patients and patients with established CVD.
